# Enhanced Inhibition of Drug-Resistant *Escherichia coli* by Tetracycline Hydrochloride-Loaded Multipore Mesoporous Silica Nanoparticles

**DOI:** 10.3390/molecules27041218

**Published:** 2022-02-11

**Authors:** Zhumiao Ye, Shaochen Wang, Yuelong Xu, Jianhao Zhang, Wenjing Yan

**Affiliations:** National Center of Meat Quality & Safety Control, College of Food Science and Technology, Nanjing Agricultural University, Nanjing 210095, China; 2020108054@stu.njau.edu.cn (Z.Y.); wsc@njau.edu.cn (S.W.); 2019108056@njau.edu.cn (Y.X.); nau_zjh@njau.edu.cn (J.Z.)

**Keywords:** mesoporous silica nanoparticles, tetracycline hydrochloride, drug-resistant *Escherichia coli*, long-term antibacterial activity

## Abstract

Drug-resistant bacterial infections exhibit a major threat to public health. Thus, exploring a novel antibacterial with efficient inhibition is urgently needed. Herein, this paper describes three types of MSNs (MSNs-FC2-R1, MSNs-FC2-R0.75, MSNs-FC2-R0.5) with controllable pore size (4–6 nm) and particle size (30–90 nm) that were successfully prepared. The MSNs were loaded with tetracycline hydrochloride (TCH) for effective inhibition of *Escherichia coli* (ATCC25922) and TCH-resistant *Escherichia coli* (MQ776). Results showed that the loading capacity of TCH in three types of MSNs was as high as over 500 mg/g, and the cumulative release was less than 33% in 60 h. The inhibitory rate of MSNs-FC2-R0.5 loaded with TCH against *E. coli* and drug-resistant *E. coli* reached 99.9% and 92.9% at the concentration of MIC, respectively, compared with the other two types of MSNs or free TCH. Modified MSNs in our study showed a great application for long-term bacterial growth inhibition.

## 1. Introduction

The number of drug-resistant bacteria has increased exponentially over the past few years due to the abuse of antibiotics in agriculture and human health services, which poses big challenges to disease prevention and control [1]. Humans could face a post-antibiotic age because of the lost efficacy in existing antibiotics, and by then, even the simplest cold could be fatal [2]. Therefore, developing new bactericides that can combat bacterial and drug-resistant bacteria with long-term efficiency is a great emergency.

Mesoporous silica nanoparticles (MSNs) are inorganic silica nanocarrier materials with tunable pore sizes from 2 to 50 nm. Because of the unprecedented advantages of high stability, large surface area, great biocompatibility, and versatile functionalization, MSNs have attracted more and more attention in the field of biomedicine, especially in the drug delivery system [3,4]. The excellent mesoporous structure with an adjustable pore size facilitates effective drug loading and controlled release. Moreover, the easily modified surface of MSNs enhances the drug’s therapeutic efficacy and reduces toxicity, which has been approved by the US Food and Drug Administration. The prominent characteristics of MSNs provide a great opportunity for antibacterial therapy.

MSNs, functionalized with antimicrobial molecules (antibiotics, amino acids, enzymes, etc.) on the external surface or loaded with antimicrobial molecules, presented a marked inhibition of common pathogenic bacteria compared with free antibacterial molecules [5]. Tetracycline hydrochloride (TCH) is a common broad-spectrum antibiotic used for the inhibition of gram-positive and gram-negative bacteria, and its inhibitory effect on gram-positive bacteria is better than gram-negative bacteria, so the resistance of gram-negative bacteria to tetracycline hydrochloride is very serious [6]. Koneru et al. demonstrated that mobile crystalline material 41 (MCM-41) loaded with TCH showed a greater inhibition on *E. coli* after 4 h compared with free TCH. However, the loading efficiency and the antibacterial activity using MCM-41 were too low to be applied in practice [7].

Herein, dendritic MSNs with three different pore sizes (4–6 nm) were synthesized by adjusting the ratio of FC2 (sodium trifluoroacetate) and CTAB (Cetyltrimethylammonium bromide) [8]. The MSNs werWe loaded with TCH for the inhibition of common *Escherichia coli* (ATCC25922) and drug-resistant *Escherichia coli* (MQ776) [9] in vitro. The physicochemical characterization of MSNs including morphology, particle size, zeta potential, pore diameter and loading and release capacity was assessed. The antibacterial activity of MSNs loaded with TCH against *Escherichia coli* and drug-resistant *Escherichia coli* was demonstrated. TEM images and the leakage amount of protein and nucleic acid were used to reveal antimicrobial mechanisms of MSNs.

## 2. Results and Discussion

### 2.1. Characterization of MSNs

**Figure 1:**
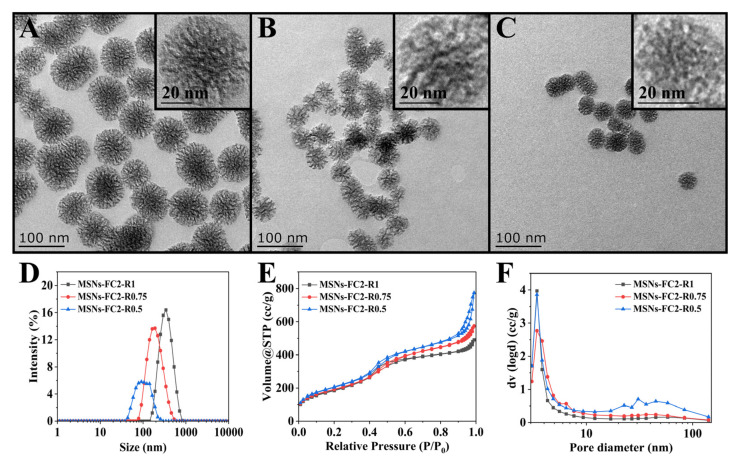
TEM images of (**A**) MSNs-FC2-R1, (**B**) MSNs-FC2-R0.75 and (**C**) MSNs-FC2-R0.5. (**D**) The particle size and (**E**) N_2_ adsorption-desorption curve and (**F**) the pore diameter of MSNs-FC2-R1, MSNs-FC2-R0.75, and MSNs-FC2-R0.5.

Figure 1A–C shows the morphology of MSNs-FC2-R1, MSNs-FC2-R0.75, and MSNs-FC2-R0.5 observed by high-resolution TEM. It was noted that three types of MSNs were spherical silica nanoparticles with large-pore dendritic structures. The average particle sizes measured by TEM were 90, 50, and 30 nm, respectively, for three types of MSNs. The hydrodynamic diameters of MSNs-FC2-R1, MSNs-FC2-R0.75, and MSNs-FC2-R0.5 measured by dynamic light scattering (DLS) were 330, 192, and 180 nm, respectively (Figure 1D), which is larger than that of TEM due to the strong surface hydration of silica [10].

To demonstrate the porous nature of MSNs, the N_2_ adsorption-desorption isotherms were performed at 77.3 K, as shown in Figure 1E. The isotherms are identified as type IV according to The International Union of Pure and Applied Chemistry (IUPAC) classification [11], indicating a characteristic of mesoporous (2–50 nm) material [12]. The three MSNs samples demonstrated an increase in the adsorbed nitrogen volume at P/P_0_ values around 0.4 and 0.9, along with the emergence of H1 type hysteresis loop, suggesting the nitrogen capillary condensation inside the mesopores (Figure 1E). The Barrett–Joyner–Halenda (BJH) method [13] was used to determine the pore size distribution (PSD) of MSNs (Figure 1F). The surface area, pore size and pore volume of three MSNs were summarized in Table 1. When MSNs were synthesized using FC2 as an additive at various R (R = 1, 0.75 and 0.5), the specific BET surface area increased from 675.936 to 740.652 m^2^ g^−1^ and the pore volume increased from 0.7609 to 1.200 cm^3^ g^−1^, as R decreased. The pore diameter was of MSNs-FC2-R1, MSNs-FC2-R0.75, and MSNs-FC2-R0.5 was measured to be 4.503, 5.152, and 6.481 nm, respectively.

In the synthesis system of FC2, CTA^+^ cations and silicate species (from TEOS hydrolysis and condensation), fluorocarbon anions can counterpart the positively charged head groups of CTA^+^ and insert into the hydrophobic part of micelles, leading to changes in hydrophobic conditions [14]. Herein, by adjusting the ratio of FC2 and CTAB, it is possible to change the structure of MSNs, and at a lower R, the surface area, pore size and pore volume of MSNs were generally higher [15].

### 2.2. Tetracycline Loading and Release

**Figure 2:**
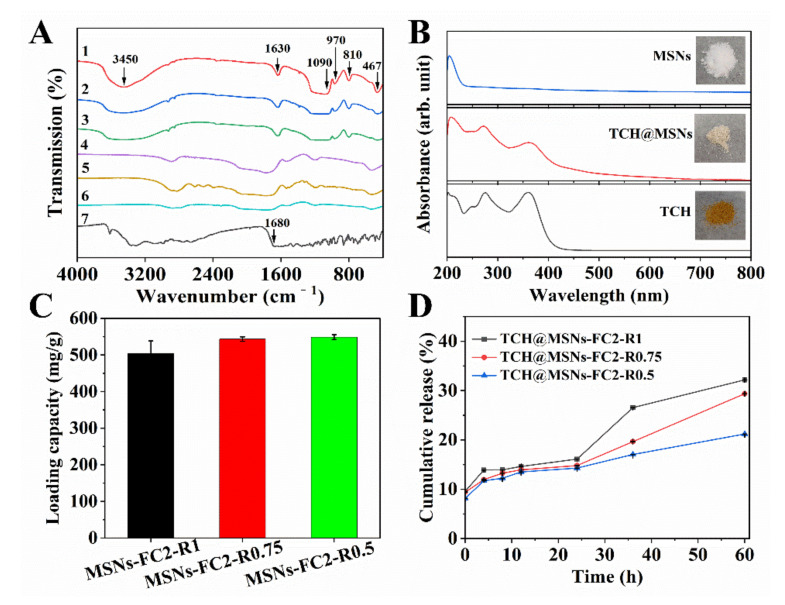
(**A**) FT-IR of (1) MSNs-FC2-R1, (2) MSNs-FC2-R0.75, (3) MSNs-FC2-R0.5, (4) TCH@MSNs-FC2-R1, (5) TCH@MSNs-FC2-R0.75, (6) TCH@MSNs-FC2-R0.5 and (7) TCH. (**B**) UV spectra and digital photos (insert) of MSNs, TCH@MSNs and TCH. (**C**) The loading capacity of MSNs-FC2-R1, MSNs-FC2-R0.75 and MSNs-FC2-R0.5. (**D**) Cumulative release of TCH@MSNs-FC2-R1, TCH@MSNs-FC2-R0.75 and TCH@MSNs-FC2-R0.5.

Fourier transform infrared spectroscopy (FT-IR) was performed to evaluate the loading of TCH on the MSNs. As shown in Figure 2A, pure MSNs showed a wide band at 1090 cm^−1^, and weak bands at 810 cm^−1^ and 465 cm^−1^, respectively, which were attributed to the asymmetric stretching vibration, symmetric stretching vibration and bending vibration of the Si–O–Si bond. The band at 970 cm^−1^ was attributed to the Si–OH bending vibration. The bands at 3450 cm^−1^ and 1630 cm^−1^ were attributed to the asymmetric stretching vibration and the H–OH bending vibration of water [16]. For pure TCH, the band at 3450 cm^−1^ was due to the first overtone of O–H bending, the peak at 1680 cm^−1^ corresponds to a C=O vibration band [17]. In the case of TCH-loaded MSNs, the band at 3450 cm^−1^, 1090 cm^−1^, 1630 cm^−1^ and 810 cm^−1^ were strongly reduced in intensity compared with MSNs. Moreover, the band at 1630 cm^−1^ was red-shifted by 120 cm^−1^ and the band at 3450 cm^−1^ was blue-shifted by 550 cm^−1^. This suggested that the new wrapped phase was formed between TCH and MSNs in the porous channels, changing the charge density [18]. The changes in the UV-Vis spectrum and the powder color of TCH-MSNs also demonstrated the successful load of TCH, as shown in Figure 2B.

The loading capacity of three types of MSNs was determined by measuring the OD value at 275 nm via UV-Vis spectrum. As shown in Figure 2C, MSNs-FC2-R1, exhibited the lowest loading capacity of 504.12 mg/g, and MSNs-FC2-R0.5 showed the highest loading capacity of 548.81 mg/g, which was attributed to the increased pore volume of MSNs-FC2-R0.5. Figure S3 (Supplementary Materials) showed the surface zeta potential of MSNs before and after TCH loading in PBS solution. We noted that the surface charges of all three MSNs showed very small increases (1–2 mV) after TCH loading, indicating that most of the TCH molecule was immobilized in the mesopores of MSNs by non-covalent interactions instead of modifying on the surface of MSNs [19,20].

TCH release behavior from MSNs was examined under conditions with a fixed initial TCH concentration (0.2 mg/mL) in PBS. As shown in Figure 2D, three MSNs showed a more sustained release profile compared to the previous report [21]. The maximum cumulative release of MSNs-FC2-R1, MSNs-FC2-R0.75 and MSNs-FC2-R0.5 was 32.2%, 29.4% and 21.2%, respectively, within 60 h. Among them, MSNs-FC2-R0.5 showed the retarded release of TCH molecules especially after 24 h, which may be related to the surface roughness and non-covalent interactions [15,22]. The drug delivery results demonstrated that the prepared three MSNs had large TCH adsorption capacities (over 500 mg/g) and sustained drug releases over 60 h, which could significantly maximize the long-term antibacterial efficacy of the drug.

### 2.3. Antibacterial Activity of MSNs

**Figure 3:**
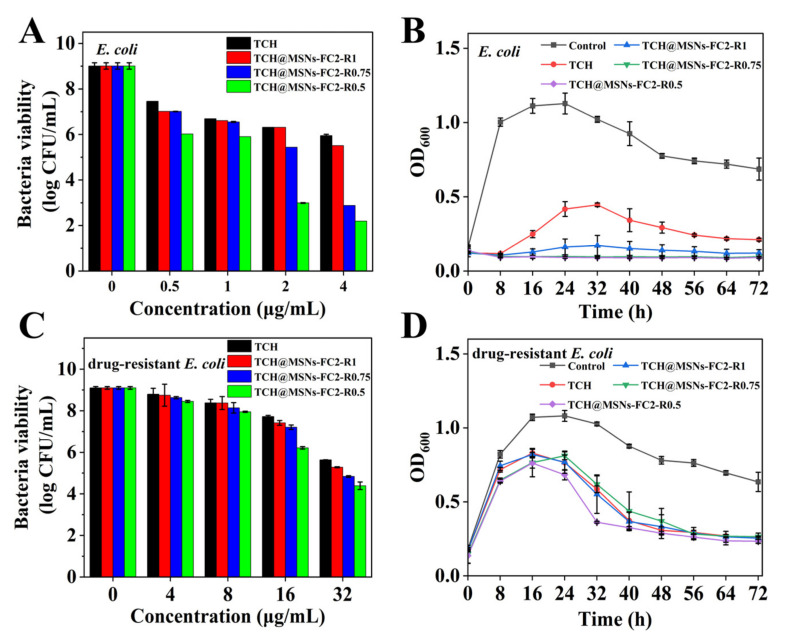
Bacteria viability of (**A**) *E. coli* and (**C**) drug-resistant *E. coli* incubated with TCH and MSNs loaded with TCH at different concentrations for 24 h. Growth kinetics of (**B**) *E. coli* and (**D**) drug-resistant *E. coli* incubated with TCH and MSNs loaded with TCH in 72 h. The concentration of TCH in (**B**) and (**D**) was 4 μg/mL and 32 μg/mL, respectively.

To assess the in vitro antibacterial activity of TCH-loaded MSNs, *E. coli* (ATCC25922) and TCH-resistant *E. coli* (MQ776) were selected as typical bacteria. TCH was chosen as the positive control group. The colony-forming units (CFUs) of bacteria after 24 h of incubation with TCH or TCH-loaded MSNs were investigated, respectively. As shown in Figure 3A,C, all groups exhibited dose-dependent antibacterial performance. We noted that more than half of the bacteria survived in the free TCH group even at the concentration of 4 μg/mL (Figure 3A). Meanwhile, TCH-loaded MSNs-FC2-R0.5 showed the lowest viability (from 9.01 log to 2.19 log) at the same concentration compared with other groups, suggesting the strongest bactericidal activity, which was consistent with the photograph of the agar plate (Figure S4, Supplementary Materials). TCH-loaded MSNs-FC2-R0.5 had the lowest MIC value of 1 μg/mL, which was lower than free TCH (MIC determined to be 2 μg/mL), as shown in Table 2. The long-term antibacterial activity of TCH-loaded MSNs was evaluated by measurement of OD_600_ value. As shown in Figure 3B, TCH-loaded MSNs-FC2-R0.5 and MSNs-FC2-R0.75 at the concentration of 4μg/mL exhibited maintained 100% inhibition towards *E. coli* throughout 72 h. The long-term bacterial inhibition property should be attributed to the larger surface area and pore volume of MSNs, which enabled more TCH to enter inside the bacterial cells [23].

For drug-resistant *E. coli*, the antimicrobial activity of TCH-loaded MSNs was not as high as in *E. coli.* Even treated at the high concentration of 32 μg/mL for 24 h, bacterial viability remains as high as 5.64 log for TCH and 4.39 log for TCH-loaded MSNs-FC2-R0.5 (Figure 3C). The photograph of the agar plate confirmed the strongest inhibition of TCH-loaded MSNs-FC2-R0.5 towards drug-resistant *E. coli* compared with other groups (Figure S5 Supplementary Materials). The MIC value of TCH-loaded MSNs-FC2-R0.5 against TCH-resistant *E. coli* was 8 μg/mL, which was 4 times lower than free TCH (MIC determined to be 32 μg/mL), as shown in Table 2. Bacterial growth kinetics demonstrated that no differences in the inhibition of bacteria were observed after 40 h between TCH-loaded MSNs-FC2-R0.5 and TCH (Figure 3D). We speculated that at the beginning of inhibition (within 40 h), more tetracycline was transported inside the bacteria, MSNs showed significant inhibition compared with free TCH, but in the later period, more TCH molecules released from MSNs were discharged by the efflux pump in drug-resistant *E. coli* [24], which results in the decrease in the antibacterial activity of TCH-loaded MSNs against drug-resistant *E. coli*.

### 2.4. Antibacterial Mechanism

**Figure 4:**
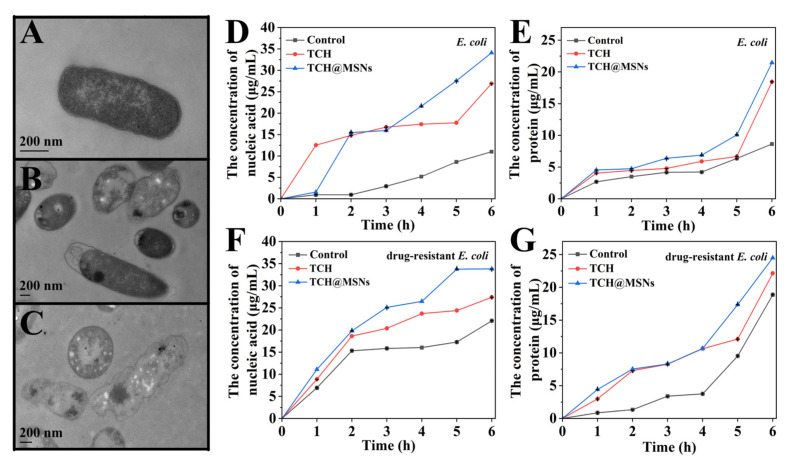
TEM images of *E. coli* after treatment with (**A**) PBS (control), (**B**) TCH and (**C**) TCH@MSNs-FC2-R0.5 for 24 h. The concentration of (**D**) nucleic acid and (**E**) protein released from *E. coli* after treatment with TCH@MSNs-FC2-R0.5 and TCH, respectively. The concentration of (**F**) nucleic acid and (**G**) protein released from drug-resistant *E. coli* after treatment with TCH@MSNs-FC2-R0.5 and TCH, respectively. The concentration of TCH in (**A**,**D**,**E**) was 4 μg/mL. The concentration of TCH in (**F**,**G**) was 32 μg/mL.

To further investigate the effect of TCH-loaded MSNs on cellular structure, TEM was employed to observe the morphology changes in *E. coli* after treatment with TCH and TCH-loaded MSNs-FC2-R0.5 (at the concentration of 4 μg/mL) for 24 h (Figure 4A–C). In comparison with untreated cells (Figure 4A), both TCH and TCH-loaded MSNs treatments disrupted the *E. coli* cell membrane, resulting in the loss of integrity of the cell membrane and the leakage of cytoplasmic contents (Figure 4B,C). Especially, more severe membrane damage and cytoplasmic leakage were observed for bacteria treated with TCH-loaded MSNs-FC2-R0.5. Some bacteria appear hollow and disfigured cell walls, indicating the serious leakage of nucleic acids and proteins, which was consistent with the results shown in Figure 4D–G. Compared with the control and TCH groups, the bacteria after treatment with TCH-loaded MSNs-FC2-R0.5 for 6 h exhibited a higher amount of leaked nucleic acids and proteins.

The enhanced antibacterial activity of TCH-loaded MSNs-FC2-R0.5 was attributed to the efficient drug delivery of MSN and the inhibition of bacterial protein synthesis of TCH. High concentration TCH can be easily transported into the cells in a short time with the help of the MSN, they can bind to the receptor on the 30S subunit of the ribosome of bacteria, preventing the elongation of the peptide chain, and inhibiting the protein synthesis. The blocking of protein synthesis further changes the permeability of the bacterial cell membrane, resulting in cell death [25].

## 3. Materials and Methods

### 3.1. Materials

Cetyltrimethylammonium bromide (CTAB), triethanolamine (TEA), tetraethyl orthosilicate (TEOS, 98%), sodium trifluoroacetate (FC2), tetracycline hydrochloride (TCH), were purchased from Sigma-Aldrich (Shanghai, China), phosphate buffer solution (PBS, 0.01 M, pH = 7.2–7.4).

### 3.2. Preparation of Modified Mesoporous Silica Nanoparticles (MSNs)

Mesoporous silica nanoparticles with adjustable pore size were prepared by tuning the molar ratios (R) of fluorocarbon anions to CTAB based on a previous report with some modifications [14]. Briefly, 380 mg of CTAB and a certain amount of FC2 were added to 25 mL ultrapure water followed by adding 68 mg of TEA, the mixture was stirred continuously for 1 h at 80 °C. Then, 1.5 mL of TEOS was added to the above-prepared mixture slowly and continuously. After the reaction was kept for 2 h, the precipitate was collected by centrifugation at 5000 rpm for 7 min (Allegra-64R Centrifuge, Beckman Coulter, CA, USA), and washed with ethanol several times. Then the precipitates were resolved in an ethanol solution of NH_4_NO_3_ (50 mL, 10 mg/mL), and incubated at 60 °C for 24 h to remove the surfactants [26]. The products were collected after centrifugation and denoted as MSNs-FC2-Rx, where x represents the molar ratio of FC2 to CTAB.

### 3.3. Tetracycline Hydrochloride Adsorption and Release Profile

Typically, 2 mg of MSNs were dispersed in 2 mL of 1 mg/mL TCH solution, the solution was stirred at room temperature for 12 h. Unloaded TCH in the supernatant was collected by centrifugation at 13,000 rpm for 10 min and determined by using ultraviolet-visible absorption spectrometry (UV-2600/2700, Shimadzu, Japan) at a wavelength of 275 nm. A serial of TCH dilutions (0.5–64 μg/mL) was prepared in PBS as the standard curve (Figure S1, Supplementary Materials). The TCH loading capacity (mg/g) of MSNs can be calculated by the following formula:

(1) $$Loading\;capacity\;\left(mg/g\right)=\frac{TCH_{total}-TCH_{supernatant}}{m_{MSNs}}$$

where *TCH_total_* is the total content of TCH in solution, *TCH_supernatant_* is the residual TCH content in the supernatant, *m_MSNs_* is the total content of MSNs in solution. In the blank control group, 2 mL of PBS (0.01 M, pH = 7.2–7.4) instead of TCH solution was mixed with 2 mg of MSNs.

The release of TCH from the MSNs solution was investigated by a dialysis method. Briefly, 5 mL of TCH loaded MSNs solution was transferred into a dialysis bag (MWCO = 14,000), which was further immersed in 100 mL of PBS solution (0.01 M, pH = 7.2–7.4) with gentle shaking at 37 °C. 1 mL of the release medium (replacing with 1 mL of fresh PBS (0.01 M, pH = 7.2–7.4) was measured by using UV-Vis at a wavelength of 275 nm. The drug cumulative release (%) of MSNs was calculated by the following formula:

(2) $$Cumulative\;release\;\left(\%\right)=\frac{CnV+\sum_{i=1}^{n-1}CiVi}{m_{TCH}}$$

where *Cn* is the TCH concentration in the sample at the nth sampling, *V* is the total volume of released medium, *Ci* is the TCH concentration in the sample at the ith sampling, *Vi* is the sample volume at the ith sampling, and *m_TCH_* is the total quality of TCH in the input system.

### 3.4. Characterization of MSNs

The morphology of MSNs was observed by transmission electron microscopy (TEM, JEM-1011, JEOL, Tokyo, Japan) at 200 kV (Cs 0.5 mm, point resolution 1.9 Å). The size and zeta potential of MSNs were obtained with Zetasizer Nano ZS90 (Malvern Instruments Ltd., Malvern, UK). Nitrogen adsorption–desorption was measured by Quantachrome Instruments version 5.21 (Compass test Zhengzhou office, Zhengzhou, China) at 77 K. The samples were degassed at 453 K overnight before the measurement. The total pore volume was calculated from the adsorbed amount at a relative pressure (P/P_0_) of 0.99. The specific surface area was calculated based on Nitrogen adsorption-desorption measurements using Brunauer–Emmett–Teller (BET) method. The pore size distribution was calculated using Barrett–Joyner–Halenda (BJH) method. The structure information of MSNs before and after TCH loading was performed by Fourier transform infrared spectroscopy (FT-IR) (Nicolet-6700, Thermo Fisher Scientific, Shanghai, China). The spectra were obtained with 4 cm^−1^ resolution, and the average value was obtained in 64 scans.

### 3.5. Antimicrobial Activity Assays

#### 3.5.1. Bacterial Culture

*E. coli* (ATCC25922) and tetracycline-resistant *E. coli* (MQ776) derived from soil samples [9] were cultured in Luria–Bertani (LB) broth at 37 °C for 7 h. The bacteria were harvested by centrifugation at 7000 rpm for 7 min, and the concentration was adjusted to approximately 10^8^ colony-forming units per milliliter (CFU/mL) by measuring the OD value at a wavelength of 600 nm.

#### 3.5.2. Determination of Minimal Inhibitory Concentration (MIC)

A total of 1 mL of the samples (TCH or TCH-loaded MSNs, including MSNs-FC2-R1, MSNs-FC2-R0.75, and MSNs-FC2-R0.5) in PBS at different TCH (0.25–64 µg/mL) concentrations were, respectively, added to *E. coli* and tetracycline-resistant *E. coli* suspension in sterile LB (1 mL of 10^8^ CFU/mL). The solution was incubated at 37 °C for 24 h. The turbidity of bacteria treated with TCH or TCH-loaded MSNs was determined by OD_600_ using a multifunctional microplate reader (Tecan infinite M200, Growth Curves Ltd., Raisio, Finland). Each concentration was prepared and measured in triplicate.

#### 3.5.3. Antibacterial Activity of MSNs In Vitro

The total plate count method was used for testing the antibacterial activity of TCH-loaded MSNs (MSNs-FC2-R1, MSNs-FC2-R0.75, and MSNs-FC2-R0.5) against *E. coli* and drug-resistant *E. coli*, respectively. Typical, bacterial suspension (1 mL of 10^8^ CFU/mL) was added to 1 mL of TCH-loaded MSNs solution (MSNs-FC2-R1, MSNs-FC2-R0.75, and MSNs-FC2-R0.5). After 24 h of incubation at 37 °C, the solutions were collected, and the 10-fold serially diluted cells were spotted onto Baird–Parker and EMB agar. CFUs were counted after incubation at 37 °C for 24 h. Given that the sensitivity of common *E. coli* and drug-resistant *E. coli* to TCH was distinct, the concentration of TCH-loaded MSNs incubated with *E. coli* was 0.5, 1, 2 and 4 µg/mL, respectively, while the concentration of TCH-loaded MSNs incubated with drug-resistant *E. coli* was 4, 8, 16 and 32 µg/mL, respectively. Meanwhile, TCH at the same concentration was used as the positive group, and saline (0.9% NaCl) was used as the control group.

#### 3.5.4. Bacterial Kinetic Activity

The bacterial kinetic activity after treatment with TCH or TCH-loaded MSNs was determined by OD_600_ reading within 72 h. Typically, 1 mL of TCH or TCH-loaded MSNs (MSNs-FC2-R1, MSNs-FC2-R0.75, and MSNs-FC2-R0.5) in PBS was added to 1 mL of bacterial suspension in LB medium (10^8^ CFU/mL), which was further incubated at 37 °C. aThe concentration of TCH-loaded MSNs incubated with *E. coli* was 4 µg/mL, and the concentration of TCH-loaded MSNs incubated with drug-resistant *E. coli* was 32 µg/mL. Each concentration was prepared and measured in triplicates.

### 3.6. Determination of the Leaked Nucleic Acid and Proteins Content

The concentration of nucleic acid leaked from *E. coli* and drug-resistant *E. coli* was determined by absorbance measurements at 260 nm (OD_260_). The bacterial suspension (10^8^ CFU/mL) was incubated with TCH or TCH-loaded MSNs-FC2-R0.5 (4 μg/mL and 32 μg/mL), respectively, at 37 °C for 6 h. The supernatant was collected every 1 h by centrifugation at 13,000 rpm for 5 min and determined the absorbance value at 260 nm. Saline was used as a control group.

The concentration of protein leaked from *E. coli* and drug-resistant *E. coli* was determined by following the Coomassie brilliant blue method. The bacterial suspension (10^8^ CFU/mL) was incubated with TCH or TCH-loaded MSNs-FC2-R0.5 (4 μg/mL and 32 μg/mL), respectively, at 37 °C for 6 h. A total of 1 mL of the supernatant was collected every 1 h by centrifugation at 13,000 rpm for 5 min and added to 4 mL of staining solution. After incubation for 5 min, the absorption was read at 595 nm. The protein content was calculated by taking bovine serum protein as the standard curve (Figure S2, Supplementary Materials). Saline was used as a control group.

## 4. Conclusions

In summary, three types of MSN (MSNs-FC2-R1, MSNs-FC2-R0.75 and MSNs-FC2-R0.5) with tunable particle diameter (30–90 nm) and pore size (4–6 nm) were fabricated by adjusting the amount of sodium trifluoroacetate (FC2). MSNs prepared in this work exhibited a high TCH loading capacity of up to 500–550 mg/g and a controlled release rate for long periods of 72 h. Among them, MSNs-FC2-R0.5 with small particle size as well as larger pore size and volume showed the strongest antibacterial activity against *E. coli* and drug-resistant *E. coli,* after TCH loading. Moreover, the MIC value of TCH-loaded MSNs-FC2-R0.5 was 4 times lower than that of TCH against drug-resistant *E. coli*, which may provide a new approach for the design of nanocarriers for the treatment of resistant bacteria.

## Figures and Tables

**Table 1:** N_2_ adsorption-desorption data of MSNs.

Sample Name	D (nm)	d (nm)	S (m^2^ g^−1^)	V (cm^3^ g^−1^)
R1	90	4.503	675.936	0.7609
R0.75	50	5.152	691.091	0.8901
R0.5	30	6.481	740.652	1.200

Note: D is the particle size, d represents the pore size, S refers to the surface area, and V indicates the total pore volume.

**Table 2:** Minimum inhibitory concentration (MIC).

Sample name	Tetracycline Hydrochloride (TCH)	TCH@MSNs-FC2-R0.5
*E. coli* (ATCC25922)	2 μg/mL	1 μg/mL
TCH-resistant *E. coli* (MQ776)	32 μg/mL	8 μg/mL

## Data Availability

Not applicable.

## Supplementary Materials

The following are available online, Figure S1. Standard curve of tetracycline hydrochloride, Figure S2. Standard curve of bovine serum albumin, Figure S3. Zeta potential of (a) MSNs-FC2-R1, (b) MSNs-FC2-R0.75, (c) MSNs-FC2-R0.5, (d) TCH, (e) TCH@MSNs-FC2-R1, (f) TCH@MSNs-FC2-R0.75 and (g) TCH@MSNs-FC2-R0.5, Figure S4. Optical pictures of *E. coli* incubated with (A) Control, (B) TCH, (C) TCH@MSNs-FC2-R1, (D) TCH@MSNs-FC2-R0.75, (E) TCH@MSNs-FC2-R0.5 at the concentration of 4 μg/mL, Figure S5. Optical pictures of drug-resistant *E. coli* incubated with (A) Control, (B) TCH, (C) TCH@MSNs-FC2-R1, (D) TCH@MSNs-FC2-R0.75, (E) TCH@MSNs-FC2-R0.5 at the concentration of 32 μg/mL.

## Sample Availability

Samples of the compounds MSNs are available from the authors.
