# Supplementary material for: Enhanced Inhibition of Drug-Resistant Escherichia coli by Tetracycline Hydrochloride-Loaded Multipore Mesoporous Silica Nanoparticles
Source: Molecules. 2022 Feb 11;27(4):1218. doi: 10.3390/molecules27041218 (PMC8877189; doi:10.3390/molecules27041218)
Supplement: Supplementary file 1 [file molecules-27-01218-s001.zip › molecules-1579487-supplementary.pdf]

Supplementary Materials

Enhanced Inhibition of Drug-Resistant *Escherichia coli* by Tetracycline Hydrochloride-Loaded Multipore Mesoporous Silica Nanoparticles

Zhumiao Ye, Shaochen Wang, Yuelong Xu, Jianhao Zhang and Wenjing Yan *

National Center of Meat Quality & Safety Control, College of Food Science and Technology, Nanjing Agricultural University, Nanjing 210095, China; 2020108054@stu.njau.edu.cn (Z.Y.); wsc@njau.edu.cn (S.W.); 2019108056@njau.edu.cn (Y.X.); nau_zjh@njau.edu.cn (J.Z.)

* Correspondence: ywj1103@njau.edu.cn; Tel.: +01-589-589-3834

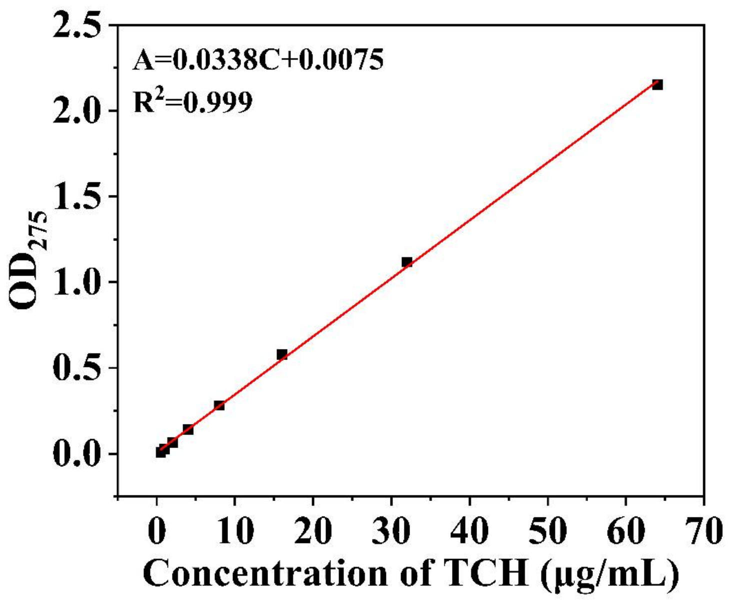

Figure S1. Standard curve of tetracycline hydrochloride.

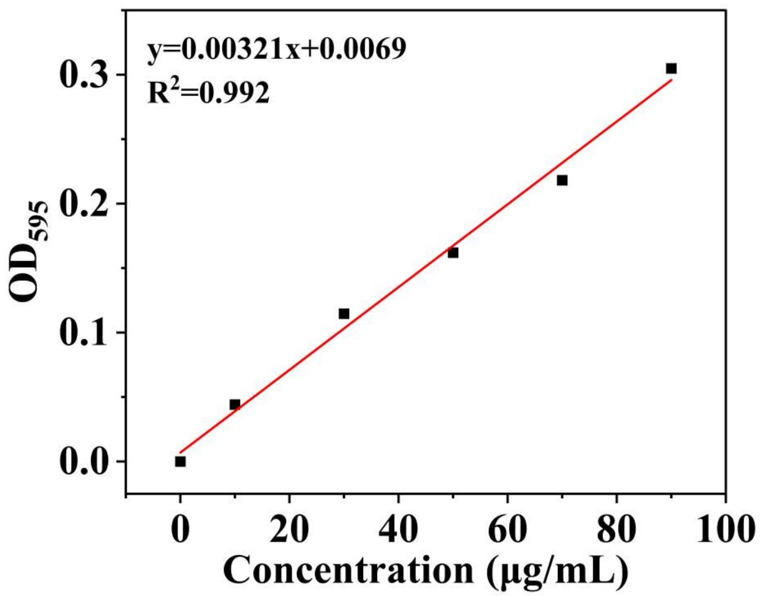

Figure S2. Standard curve of bovine serum albumin.

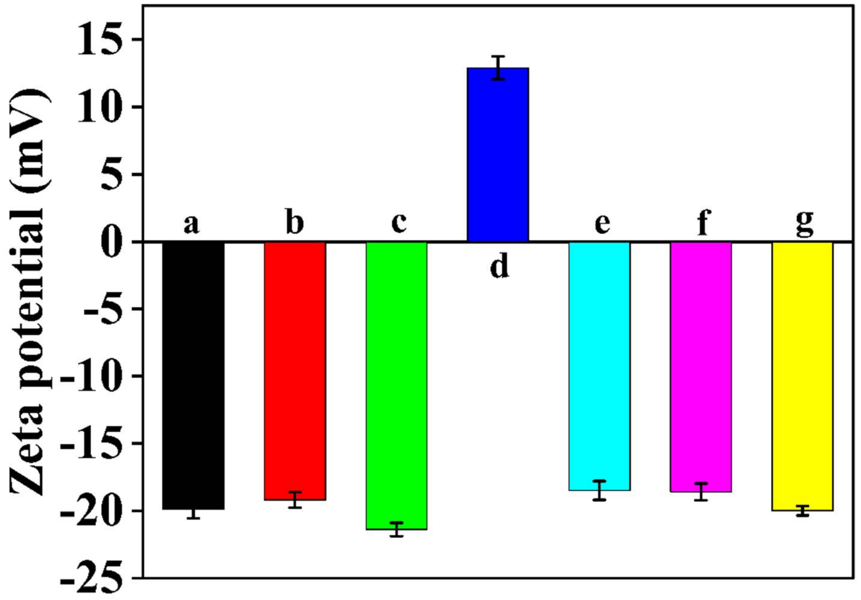

Figure S3. Zeta potential of (a) MSNs-FC2-R1, (b) MSNs-FC2-R0.75, (c) MSNs-FC2-R0.5, (d) TCH, (e) TCH@MSNs-FC2-R1, (f) TCH@MSNs-FC2-R0.75 and (g) TCH@MSNs-FC2-R0.5.

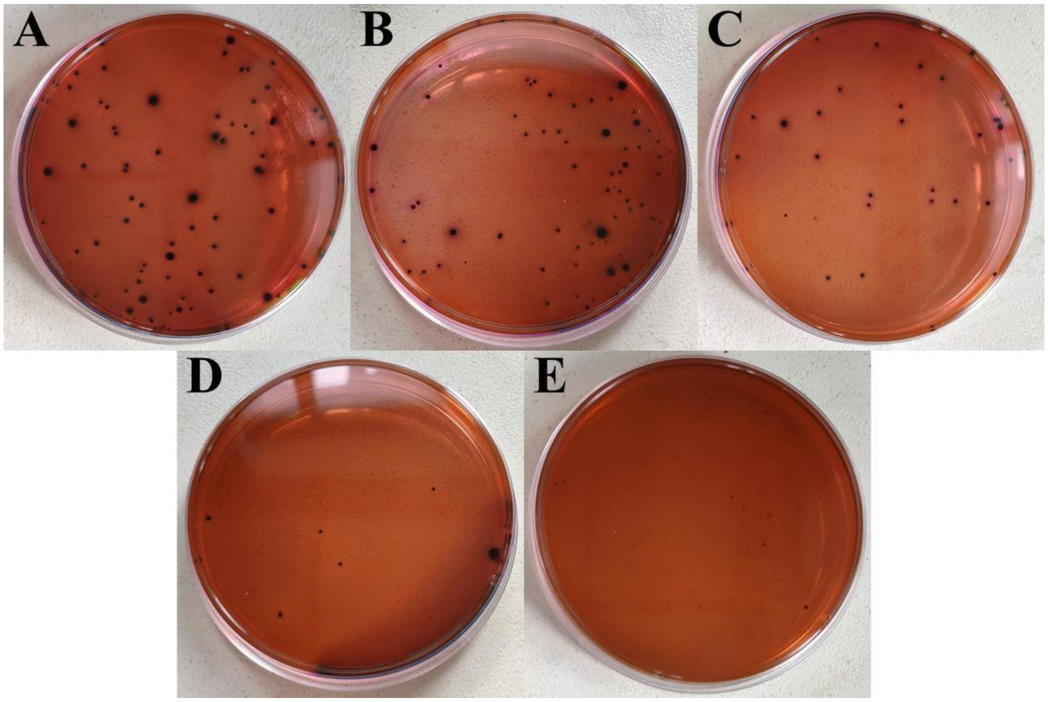

Figure S4. Optical pictures of *E. coli* incubated with (A) Control, (B) TCH, (C) TCH@MSNs-FC2-R1, (D) TCH@MSNs-FC2-R0.75, (E) TCH@MSNs-FC2-R0.5 at the concentration of 4 μg/mL.

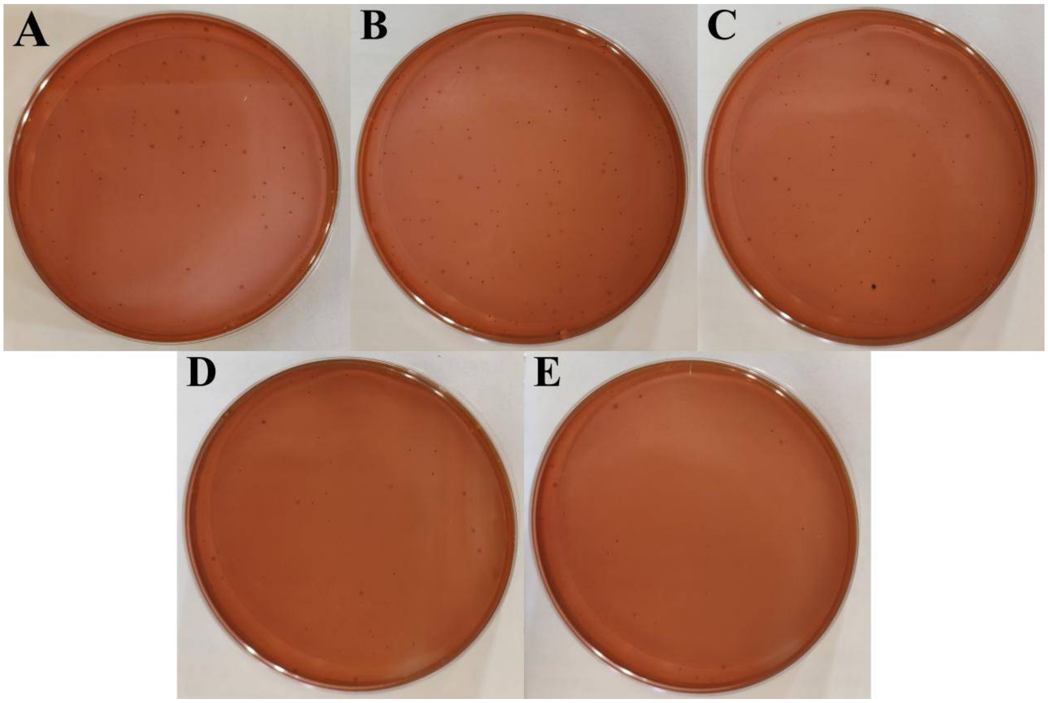

Figure S5. Optical pictures of drug-resistant *E. coli* incubated with (A) Control, (B) TCH, (C) TCH@MSNs-FC2-R1, (D) TCH@MSNs-FC2-R0.75, (E) TCH@MSNs-FC2-R0.5 at the concentration of 32 μg/mL.
